# rRNA expansion segment 27Lb modulates the factor recruitment capacity of the yeast ribosome and shapes the proteome

**DOI:** 10.1093/nar/gkaa003

**Published:** 2020-01-21

**Authors:** Vaishnavi Shankar, Robert Rauscher, Julia Reuther, Walid H Gharib, Miriam Koch, Norbert Polacek

**Affiliations:** 1 Department of Chemistry and Biochemistry, University of Bern, Freiestrasse 3, 3012 Bern, Switzerland; 2 Graduate School for Cellular and Biomedical Sciences, University of Bern, Bern, Switzerland; 3 Interfaculty Bioinformatics Unit, University of Bern, Baltzerstrasse 6, 3012 Bern, Switzerland

## Abstract

Fine-tuned regulation of protein biosynthesis is crucial for cellular fitness and became even more vital when cellular and organismal complexity increased during the course of evolution. In order to cope with this augmented demand for translation control, eukaryal ribosomes have gained extensions both at the ribosomal protein and rRNA levels. Here we analyze the functional role of ES27L, an rRNA expansion segment in the large ribosomal subunit of *Saccharomyces cerevisiae*. Deletion of the b-arm of this expansion segment, called ES27Lb, did not hamper growth during optimal conditions, thus demonstrating that this 25S rRNA segment is not inherently crucial for ribosome functioning. However, reductive stress results in retarded growth and rendered unique protein sets prone to aggregation. Lack of ES27Lb negatively affects ribosome-association of known co-translational N-terminal processing enzymes which in turn contributes to the observed protein aggregation. Likely as a compensatory response to these challenges, the truncated ribosomes showed re-adjusted translation of specific sets of mRNAs and thus fine-tune the translatome in order to re-establish proteostasis. Our study gives comprehensive insight into how a highly conserved eukaryal rRNA expansion segment defines ribosomal integrity, co-translational protein maturation events and consequently cellular fitness.

## INTRODUCTION

The ribosome is a large, complex ribonucleoprotein (RNP) assembly that is responsible for protein biosynthesis in all domains of life. It is highly conserved at its central functional cores such as the decoding center and the peptidyl transferase center (PTC). The basic mechanism of translation shares common features throughout the three domains of life and is also divided into the same sub-steps (initiation, elongation, termination and recycling). However, the eukaryotic ribosomes have additional functions, such as mRNA scanning during translation initiation and frequent nascent chain translocations through organellar membranes. To cope with these expanded tasks, eukaryal ribosomes have evolved sophisticated layers of translational regulation. One of the striking differences between prokaryal and eukaryal ribosomes is the size – over the course of evolution the ribosome has expanded in response to the increasing functional and regulatory demands of the cell, whilst conserving the central core. These expansions have occurred in both subunits of the ribosome, in both the rRNA and associated ribosomal proteins (RP). Prokaryotic ribosomes consist of a large (50S) subunit, comprised of the 5S and 23S rRNAs and 33 r-proteins and a small (30S) subunit containing one 16S rRNA and 21 r-proteins. The two subunits together constitute the 2.5-megadalton (MDa) 70S ribosome. Their eukaryotic counterparts are ∼40% larger in size with the 60S and 40S subunits forming the 80S ribosome (their molecular weights ranging from 3.5 MDa to 4.5 MDa). The small subunit of the eukaryotic ribosome (40S) contains 18S rRNA and 33 r-proteins, while the large subunit (60S) contains 5.8S, 25S (or 28S in multicellular eukaryotes), 5S rRNAs and 46–47 r-proteins (1). The additional nucleotide sequences on the rRNA are called expansion segments (ES) and insertions appear mostly at the periphery of the ribosome. This increase in size from single cellular life to more complex eukaryotes is predicted to be due to additional functions that the ribosome has to carry out, such as export from the site of biogenesis within an enclosed nucleus, anchorage to membranes of organelles, or additional layers of co- and post- translational controls that are enforced in a more complex cell. The ES insertions in the rRNA range in size from eight nucleotides (nts) to 150 nts (in length) in yeast, and even much longer in mammals (2). Remarkably though, apart from an increase in the number of nucleotides, the insertion sites of the ES are conserved in all eukaryotes (3,4). While their location and accessibility on the surface of the ribosome suggest possible involvement in binding of auxiliary factors, the exact biological function of the various ES in translation is mostly unclear.

Attempts to understand the functionality of ES have mostly been done in yeast, and have focused on the identification of auxiliary proteins that bind to certain ES or the consequences of deletion of particular ES (5,6). Knorr *et al.* uncovered the b-arm of one of the largest ES in the 60S subunit, called ES27Lb (Supplementary Figure S1), as interaction site of the N-terminal acetyl transferase (Nat) complex with the yeast ribosome suggesting a role in co-translational acetylation of nascent peptides (5). Fujii *et al.* reported a deletion analysis of ES27Lb from the yeast ribosomes (7). Their data suggest an involvement of this ES in translation control, where they stipulate that ES27Lb coordinates binding of methionine amino peptidases (MetAP) and affects translation fidelity. Additional structural observations in both yeast and mammals show ES27L to play a role in co-translational protein translocation. In a structure of yeast 80S ribosome in complex with Sec61 (component of yeast SRP-dependent translocon complex), the ES27L b-arm was shown to be flipped in the ‘out’ position (toward L1 stalk; ES27Lb^out^), but turned toward the exit tunnel in the absence of Sec61, confirming previous modeling predictions (8). Likewise, structure of mammalian 80S localized on an endoplasmic reticulum (ER) membrane showed a density corresponding to the ES27Lb arm as interacting closely with the membrane (9). Another study on the ES involved similar deletion strategies, but it explored the involvement of ES in ribosome biogenesis (10). They report various precursor ribosomal particles accumulating in the cell upon ES27L deletion from the yeast 25S rRNA (10). These findings are in concert with the hypothesis that ES could play a role in ribosome synthesis (11–14).

In this study, by using a comprehensive approach that aims at understanding the cellular role of ES27L in relation to cellular fitness and in shaping the translatome, we explore the consequences of deleting the b-arm of the ES27L from the yeast ribosome. To the best of our knowledge, this is the first time such an approach has been conducted to elucidate in parallel the molecular role of an ES for ribosome functioning, its consequences on the complete translatome and on the cellular fitness level. Upon ES27Lb deletion, mutant ribosomes retain unaltered levels in global translational activities, yet show preferred as well as hindered translation of certain mRNAs, resulting in an altered translatome. Additionally, yeast cells harboring a pure population of mutant ribosomes show higher propensity to form protein aggregates particularly during reductive stress. This indicates potential problems in protein folding in cells that depend on ribosomes lacking ES27Lb. From our data, we conclude that ES27Lb is required for proper and coordinated co-translational nascent chain modification and, consequently, shaping the functional proteome.

## MATERIALS AND METHODS

### Yeast growth and spot assays

Yeast strain and plasmid system used was kindly provided by J. Dinman (yJD1045: (NOY891) *MATa ade2–1 ura3–1 leu2–3 his3–11 trp1 can1–100 rdn1ΔΔ::HIS3* carrying plasmid pNOY353: *35SrRNA::Trp1*) (15). All ES mutations were made on plasmid pJD694, which is a 2μ plasmid with a *URA3* selectable marker, a 5S rRNA gene under control of its endogenous RNA polymerase III promoter, and a 35S pre-rRNA operon under control of the RNA polymerase II driven, tetracycline (doxycycline) repressible TET promoter (see Supplementary Table S1 for all DNA oligonucleotides used). In addition pJD694 carries a point mutation in 18S rRNA rendering the ribosomes resistant to Hygromycin B. The mutant plasmid was transformed and used to replace the wildtype plasmid (pNOY353) in SC-Ura selection media containing Hygromycin B (15). Cells were grown and maintained at 30°C, in YPD [1% (w/v) yeast extract, 2% (w/v) Tryptone, 2% (w/v) glucose], with additional 300 μg/ml of Hygromycin B. Spot assay was performed by adjusting the yeast cultures to the same optical density of OD_600_ ∼0.5, followed by a 5-fold serial dilution, and spotting on various agar plates. The concentration of various compounds in agar plates (or where specified, in liquid cultures) was as follows: hygromycin B: 300 μg/ml; DTT: 8 mM or 16 mM or 32 mM; tunicamycin: 1 mg/ml and 2 mg/ml; paraquat: 1.5 mM and 2 mM; ethanol: 1%; cycloheximide: 100 μg/ml. For plates with a changed sugar source, complete medium plates [1% (w/v) yeast extract, 2% (w/v) tryptone] were prepared containing either 2% glucose (YPD) or 2% galactose (YPGal) or 3% glycerol (YPG). In order to generate gene knockouts, genomic DNA was isolated from *map1Δ*, *nat1Δ*, *phb1Δ*, *phb2Δ* or *hlj1Δ* cells (*Euroscarf*, each containing the G418 resistance cassette), respectively, and the respective knockout cassettes flanked by 500 bp homologous regions to the wildtype genomic locus were amplified followed by transformation of the PCR products into NOY891 carrying pJD694 with a wildtype 35S rDNA locus. Genes were replaced by homologous recombination and G418 resistant clones were used for downstream experiments.

### RNA isolation and Morgan analysis

RNA was extracted from cells by the hot phenol method as previously described (16) with the following changes: 50 ml yeast cultures in YPD + Hyg B were grown to OD_600_ = 0.8. To assess the purity of mutant rRNA, Morgan analysis was carried out as previously described (17) with the following modifications: primer 5′- TCTACAAGAGACCTACC -3′ was 5′-labeled with ^32^P, added along 0.5 μg of total RNA in a reverse transcription reaction containing 66.7 μM of ddTTP and 833 μM of the other three nucleotides as dNTPs (dATP, dGTP and dCTP), using AMV reverse transcriptase (Promega). The reaction was resolved on a 15% polyacrylamide-urea gel and visualized using a Typhoon FLA1000 phosphoimager to detect the wildtype and mutant cDNA products.

### Polysome profiling and ribosome pelleting

Yeast cells were grown in YPD at 30°C to OD_600_ = 0.8, and harvested by centrifugation at 4°C. For monitoring polysomes of DTT stressed cells, yeast cells grown to OD_600_ = 0.8 were subjected to 16 mM DTT stress for 15 min before harvesting. The cell pellets were resuspended in ice cold lysis buffer [20 mM HEPES/KOH pH 7.4, 100 mM potassium acetate, 2 mM magnesium acetate, 0.5 mM DTT, 1 mM phenylmethanesulfonyl fluoride (PMSF), 100 μg/ml cycloheximide, 1× Complete protease inhibitor cocktail (Roche)] and opened using bead disruption (FastPrep24). Lysates were cleared twice by centrifuging at 16 000 × g at 4°C for 10 min, and their absorbance values at 260 nm (*A*_260_) were determined. 20 *A*_260_ units of lysates were loaded on a 10–40% sucrose gradient (prepared in lysis buffer without PMSF), centrifuged in SW-41 tubes and rotor (Beckmann) at 39 000 rpm, for 2 h and 15 min at 4°C. After centrifugation, the gradients were fractionated and fractions corresponding to the polysomal peaks were pooled for RNA isolation as described above. To perform proteome analysis of 80S and polysomes, peaks corresponding to either single 80S peak (monosomes), light polysomes (second and third peaks) or heavy polysomes (beyond third peaks) were all collected separately and pelleted by centrifuging at 100 000 × g for 15 h at 4°C in an SW-41 rotor. Pellets were directly provided for mass spectrometric analysis. Mass spectrometric analysis of whole cell lysates was performed on TCA precipitated proteins derived from cells opened as described. Fold-changes were calculated as ratios of ΔES27Lb over wildtype levels. This ratio was log_2_-transformed to yield log_2_-fold changes.

### Metabolic labeling

Cells were grown in complete media (YPD) to OD_600_ = 0.7, and five OD_600_ units of cells were harvested by pelleting at 2500 × g for 2 min at room temperature. Newly synthesized proteins were labeled in methionine-free medium supplemented with 15 μCi/ml ^35^S l-methionine for a 5 ml reaction, and incubated at 30°C with rotation for 1 h. For metabolic labeling in the presence of DTT, 16 mM of DTT (f.c.) was added to the reaction before incubation. At each time interval required, 500 μl of labeled culture was removed, and the reaction stopped by the addition of 100 μg/ml clycoheximide (CHX). Subsequently, the cells were pelleted at 2500 × g for 2 min at room temperature. The cells were chased with 500 μl of ice-cold chase medium (SC-Met, 50 μg/ml methionine, 0.5 μg/ml CHX) and pelleted again as before. The proteins were precipitated with 20% TCA and loaded on 10% SDS gel, and radioactive signals scanned and quantified using the TyphoonFLA1000 Phosphoimager.

### Aggregate isolation

Protein aggregates were isolated as described (18), but with the following changes: Cells were grown in YPD at 30°C to OD_600_ = 0.8 and then DTT was added to a f.c. of 16 mM and cells were further incubated at 30°C for 15 min. The lysates prepared in the lysis buffer (20 mM KH_2_PO_4_ pH 6.8, 10 mM DTT, 1 mM EDTA pH 8.0, 0.1% Tween, 2× Easy cOmplete™ protease inhibitor (Roche), 2 mM PMSF) were sonicated in a water bath Bioruptor^®^ ultrasonicator (4× of 20 s ON/20 s OFF cycles, level setting: H). Lysates were then passed through a 23G filter needle eight times, and cell debris was pelleted at 500 × g for 5 min (4°C). The protein lysates were diluted to 4 mg/ml in 800 μl volume. The remaining lysate was pelleted at 16 000 × g, for 20 min at 4°C. Pellets were washed in wash buffer I (20 mM KH_2_PO_4_ pH 6.8, 2% NP40 (Sigma), 1× Easy cOmplete™ protease inhibitor (Roche), 2 mM PMSF) and sonicated using the water bath (4 × 15 s ON/15 s OFF, level setting: H) and then aggregates were pelleted again as before. A second wash with wash buffer I was carried out and the solution was sonicated again (3 × 15 s ON/15 s OFF, level setting: M), followed by pelleting of aggregates as before. The pelleted aggregates were washed in wash buffer II (20 mM KH_2_PO_4_ pH 6.8, 1× Easy cOmplete™ protease inhibitor (Roche), 2 mM PMSF) and sonication was performed for a final time (3 × 10 s ON/ 5 s OFF, level setting: L), followed by pelleting as before. The resulting pellet of protein aggregates was dissolved directly in 1× SDS loading buffer and all of the protein aggregates formed were resolved on a 10% SDS gel. Gels were either stained with Coomassie staining solution or gel regions were cut and proteins eluted and subjected to mass spectrometric analysis. Proteins which occurred uniquely in wildtype or ΔES27Lb and those which were more than two-fold enriched in the aggregate preparation were considered specifically aggregation prone.

### mRNA-Seq analyses

One microgram of total RNA and 1 μg polysome-associated RNA, isolated from polysome profile fractions, were poly(A) enriched and a cDNA library was prepared and sequenced using standard protocols for Illumina HiSeq3000. Sequenced raw reads were aligned to yeast strain BY4742 (reference strain) using HISAT2 (19). The reads aligned with a high rate (∼95%) to the reference genome and these reads of genomic features were counted (featureCounts) (20) and processed in R software for further analyses. The reads were normalized using the DESeq2 software (21) and translation efficiency was calculated using the differential expression method of DESeq2 contrasting polysomal mRNA pools (P) and total cellular pool of mRNAs (T). For each mRNA in a particular strain (either wildtype or mutant), a P/T ratio of reads was calculated, followed by a log_2_ transformation of these ratios. P/T ratios were then compared between wildtype and mutant highlighting condition-specific translation features. A negative P/T ratio indicated that the corresponding mRNA was poorly translated while a positive ratio indicated enhanced translation.

### qRT-PCR

Reverse transcription (RT) reactions of total and polysome-isolated RNA were carried out using SuperScript™ IV One-Step RT-PCR System (Invitrogen), according to the manufacturer's protocol, using oligo-d(T) primers for cDNA amplification. Subsequent quantitative PCR (qPCR) on the cDNA was carried out using GoTaq^®^ qPCR Master Mix, according to the manufacturer's protocol (Supplementary Table S1 lists the used primers). The QIAgility robot was used to pipette all the reagents into the qPCR reactions and qPCR amplification was carried out using Rotor Gene 6000, according to manufacturer's instructions. qPCR analyses were done in Roboticx software, and differential mRNA transcript abundances were calculated using the ΔΔC^t^ method as described (22), using an average of *GCN4, ACT1* and *TAF10* mRNA levels as house-keeping genes, as described for yeast (23).

### Northern blotting

To detect rRNA precursors, 20 μg of total RNA was loaded and separated on a 1% denaturing agarose gel and transferred to a H^+^ HYBOND nylon membrane by passive blotting, as described elsewhere (24) with the following modification: the passive blotting was carried out over 24 hrs. The membranes were hybridized using ^32^P-labeled probes for ITS1 (FL185: GGCCAGCAATTTCAAGTTA), 3′ ETS (TCCTGCCAGTACCCACTT) and ITS1 upstream of the A2 cut (CGGTTTTAATTGTCCTA), as described in (24). An autoradiogram was developed using Typhoon FLA1000 phosphoimager.

### Mass spectrometric analysis

Trypsinized peptides were detected by LC-MS using a Fusion Lumos ETD connected to a nano-UPLC column. Peptide intensities were quantified by MaxQuant LFQ algorithm (25). To address Nat complex specificity, the proteins which specifically aggregated were stratified for their first amino acid, which defines the specificity of Nat (N-terminal acetyltransferase) complexes (26). The number of aggregated proteins in each stratum was divided by the overall number of aggregated proteins.

## RESULTS

### ΔES27Lb strain is more sensitive to DTT than wildtype cells

**Figure 1.**
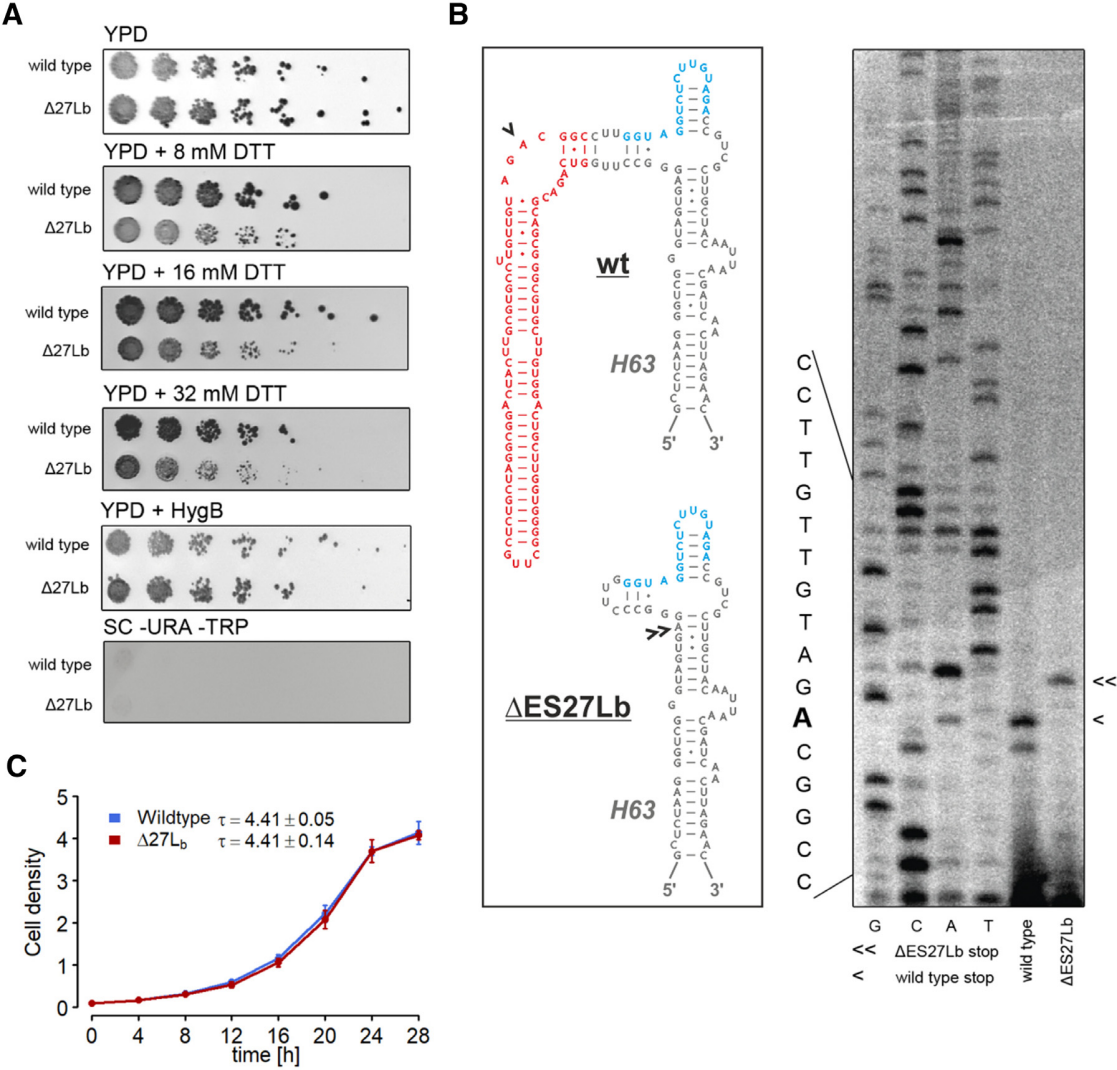
Phenotypic characterization and confirmation of ΔES27Lb mutant ribosomes. (**A**) Cells transformed with pJAD694 carrying the wildtype or ΔES27Lb rRNA sequence were analyzed via spot assays (1/5 serial dilutions). The cells were plated on YPD, YPD + hygromycin B, YPD Glu + DTT. Cells were also plated on a control plate (SC–URA-TRP) to verify loss of the original wildtype rDNA containing plasmid pNOY353. Plates were incubated for 3 days at 30°C. (**B**) The purity of the ΔES27Lb ribosome population was assessed by a modified primer extension analysis (Morgan analysis). Isolated total RNA was reverse transcribed with a dNTP/ddNTP mix ensuring a primer extension stop at the first encountered adenosine on the template rRNA. The arrow heads indicate the specific stops for the ΔES27Lb mutant and the wildtype rRNA, respectively. G, C, A, T denote a standard sequencing reaction on wildtype rRNA. The secondary structure of helix 63 (H63) and its expansion segment (red) are shown on the left for the wildtype (wt) and ΔES27Lb construct. The binding site for the used RT primer is shown in blue and the first adenosines encountered during primer extension in both constructs are denoted by arrow heads. (**C**) Wildtype (blue) and ΔES27Lb (red) cells were diluted to OD_600_ = 0.05 and growth in liquid media (YPD + Hyg B) at 30°C was monitored. The inlay gives the doubling time presented as mean ± SD deducted from three independent biological replicates.

In order to advance our understanding of the biological roles of rRNA expansion segments for ribosome functioning, we generated six individual ES truncations or deletions in *Saccharomyces cerevisiae* 18S or 25S rRNAs. The deletions resulted in different, yet specific phenotypes ranging from basically unaltered viability, to apparent growth defects, to lethality. In the first wave of experiments we focused on ES deletions that showed no or only mild growth defects. Those constructs likely give rise to mature ribosomal particles amenable to functional characterization as compared to ES deletions that would severely interfere with ribosome biogenesis. Among the introduced deletions, larger truncations such as the complete removal of ES27L or ES7L were lethal confirming previous reports of ES27L being essential for survival (7,27). To further characterize ES27L, a partial deletion was made of just the b-arm of ES27L (Supplementary Figure S1), a construct we refer to as ΔES27Lb. This arm was shown to be extremely flexible (28), with two distinct conformations (ES27L^in^ and ES27L^out^), localizing either close to the nascent chain exit tunnel or near the L1 stalk of the large ribosomal subunit. Deletion of the b-arm alone did not perturb growth in YPD media as seen from the spot assay (Figure 1A). The mutant 25S rRNA was expressed from a plasmid (pTeT) under the control of Tet_r_ (with Ura as auxotrophic marker) in a yeast strain lacking all chromosome-encoded rDNA gene copies (see Material and Methods for details). The plasmid-encoded 18S rRNA also carried a Hygromycin B (HygB) resistance point mutation to ensure stringent selection for ribosomes carrying exclusively plasmid-borne rRNA transcripts (15). The inability to grow on media lacking tryptophan shows that the cells have lost pNOY353, the plasmid carrying the wildtype rDNA with Trp as auxotrophic marker (Figure 1A). To verify that the entire population of ribosomes in the ΔES27Lb strain indeed originate from the pTET plasmid after plasmid exchange and as a consequence truly lacks the expansion segment ES27Lb, a modified primer extension reaction (Morgan analysis) was performed on total RNA isolated from the cells grown in YPD + HygB. Indeed, we observe a pure population of ΔES27Lb ribosomes and no traces of wildtype ribosomes (Figure 1B). Growth curves in full media (YPD) showed identical doubling times for the deletion mutant as compared to cells expressing the wildtype rRNAs from a plasmid (Figure 1C). The cells were also tested for growth under different physiological stress conditions to understand if ES27Lb plays a role in stress adaptation. Among the various stress conditions tested (changing sugar source, ER stress, mitochondrial stressors, redox stressors; see Supplementary Figure S2A), the ΔES27Lb cells showed a slight but reproducibly reduced growth phenotype in the presence of the strong reducing agent dithiothreitol (DTT) (Figure 1A). DTT causes reductive stress and prevents disulfide bond formation in proteins, and within the ER, such stress conditions usually activate the unfolded protein response (UPR) pathway (29). To rule out a generic ER stress or a mitochondrial stress, the cells were additionally grown in the presence of either tunicamycin (ER stress) or paraquat (mitochondrial stress). The ES deletion mutant cells grew similar to the wildtype control in both conditions, confirming that there is no generic stress to the organelles (Supplementary Figure S2A). An additional test for ER stress is to measure the level of *HAC1* mRNA splicing. *HAC1* mRNA exists as a ‘pre-mRNA’ in the cytoplasm, which does not give rise to a functional translation product. However, upon ER stress, the membrane bound endonuclease Ire1 oligomerizes and splices *HAC1* pre-mRNA in a unique splicing mechanism (30). Spliced *HAC1* mRNA is then actively translated to the transcription factor that activates further genes downstream of the UPR pathway (31). RT-PCR on mRNA isolated from these two strains showed that there was no significantly increased *HAC1* mRNA splicing under normal growth conditions in the case of the mutant compared to wildtype (Supplementary Figure S2B).

### Polysomes of the ΔES27Lb strain are similar to wildtype

**Figure 2.**
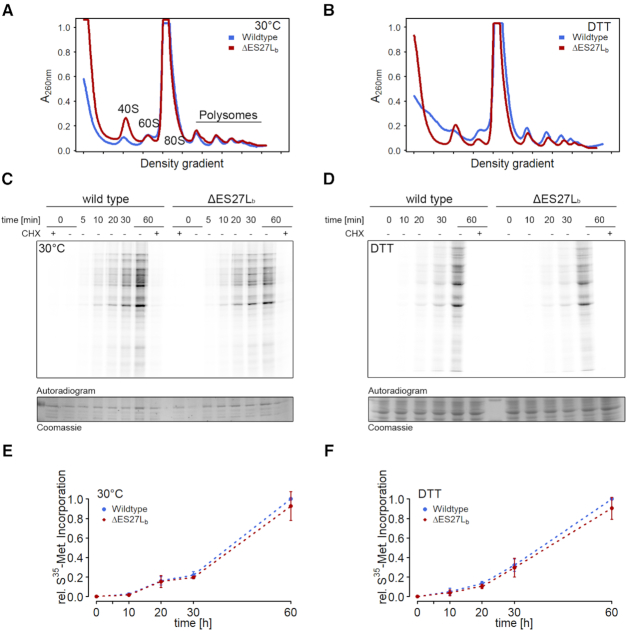
Translation status of the ΔES27Lb strain under normal conditions and during reductive stress. (**A**) Polysome profiling on sucrose density gradients of cell lysates harvested at OD_600_ = 0.7 from cells grown in YPD medium (wildtype: blue; ΔES27Lb: red). (**B**) Same as in (A) but cells were harvested at OD_600_ = 0.7 in YPD medium after a 15 min incubation with 16 mM DTT. (**C**) Metabolic labeling of newly synthesized proteins in wildtype (left) and ΔES27Lb (right) cells. The indicated time describe the pulse duration for each sample. CHX indicates whether or not cells were incubated in the presence of cycloheximide. The autoradiogram was screened with a phosphorimager whereas the loading control is derived from Coomassie stained total protein. (**D**) Metabolic labeling was performed as in (C) but in the presence of 16 mM DTT. (**E**, **F**) Quantification of ^35^S-Met incorporation during metabolic labeling experiments as depicted in (C) and (D), respectively. Data is shown as mean ± SD from three or four independent biological replicates.

Having secured an experimental yeast system ensuring a pure cellular population of mutant ribosomes, the next question was to understand how the lack of a large portion of ES27L confers no phenotype under physiological growth conditions but affected cell growth under reductive stress condition. Polysome profiles were recorded to understand the translational status of these mutants under optimal growth conditions and also under DTT stress. The results obtained indicate that ΔES27Lb ribosomes are processed and exported out of the nucleus intact and engage in active cellular mRNAs translation (Figure 2A). Similarly, under DTT stress, despite the observed growth phenotype (Figure 1A), there was no obvious difference in polysomes between mutant and wildtype (Figure 2B). There is a general reduction in translating polysomes during DTT stress, as reported earlier (32), but this reduction is similar in both the wildtype and the ΔES27Lb mutant strains. In both conditions, however, profiles for the ΔES27Lb strain show a changed subunit ratio—less 60S than 40S—compared to the wildtype control (Figure 2A, B). A previous report showed that the complete ES27L deletion results in severe ribosome biogenesis defects (10). Therefore we explored if the changed subunit ratio could be a result of improper rRNA processing in the ΔES27Lb strain. However, despite the changed subunit ratios on the polysome profile, deleting only the b-arm of the ES27L does not dramatically reduce rRNA steady state levels (Supplementary Figure S3A, B). These findings are in line with virtually unaltered polysomal fractions which represent the actively translating 80S ribosomes (Figure 2A). These data demonstrate that removing ES27Lb from 25S rRNA does not markedly affect the available pool of translationally competent ribosomal particles. To further ensure that ribosome biogenesis is not severely perturbed in the ΔES27Lb strain, we investigated the accumulation of immature rRNA species (Supplementary Figure S4A-C). A slight increase in precursor levels was observed in the deletion strain for different premature rRNA species (Supplementary Figure S4B, C). Of note, not only were precursors enriched that will eventually give rise to the mature 25S rRNA (35S rRNA and 27S rRNA; see Supplementary Figure S4A for probe designs) but also the precursor of the small subunit rRNA (20S rRNA). Since the large subunit rRNA precursors are at that stage no longer covalently connected to the 20S rRNA, argues for a general, probably indirect, decrease in rRNA maturation which however is not limited to the rRNA species comprising the ES deletion. To understand the kinetics of translation of the mutant ribosomes, the rate of protein biosynthesis was measured by monitoring ^35^S-methionine incorporation *in vivo* over time in the absence (Figure 2C, E) and presence of DTT (Figure 2D, F). Metabolic labeling activities measured for ΔES27Lb showed very similar translational rates compared to that of the wildtype strain (Figure 2C–F). Taken together, polysome profiling and metabolic labeling experiments revealed unaltered global translation rates.

### Protein composition of ribosomes lacking ES27Lb

In order to detect any possible difference in proteins bound to the mutant ribosome, mass spectrometry (MS) of translating ribosomes (polysomes) was carried out. When we focused on ribosomal proteins (RPs) which might be differentially recruited to the ribosome due to the absence of ES27Lb, we found most proteins to be present at the ribosome to comparable degrees in wildtype and ΔES27Lb ribosomes (Supplementary Figure S4D). When we quantified global levels of RP, we found a significant down shift of RP from the large subunit (RPL) in ΔES27Lb yeast (Supplementary Figure S4D), which is consistent with the reduced number of large subunits (Figure 2A). As we hypothesized altered protein binding to the ribosome, we next expanded our analysis to non-ribosomal proteins interacting with the ribosome. A GO-Term analysis of proteins depleted or enriched from translating ribosomes yielded one cluster of significantly down-regulated proteins in the ΔES27Lb strain, which contained proteins constituting the methionine amino peptidases Map1 and Map2 and the N-terminal acetylation complex NatA (Supplementary Table S2) These results are in agreement with very recently published data (5,7). Our findings suggest reduced co-translational N-terminal acetyl transferase activity in ribosomes lacking the b-arm of ES27L, a conclusion supported by recent structural data (5).

### Translatome analysis of ΔES27Lb shows preferential translation of specific sets of mRNAs

**Figure 3.**
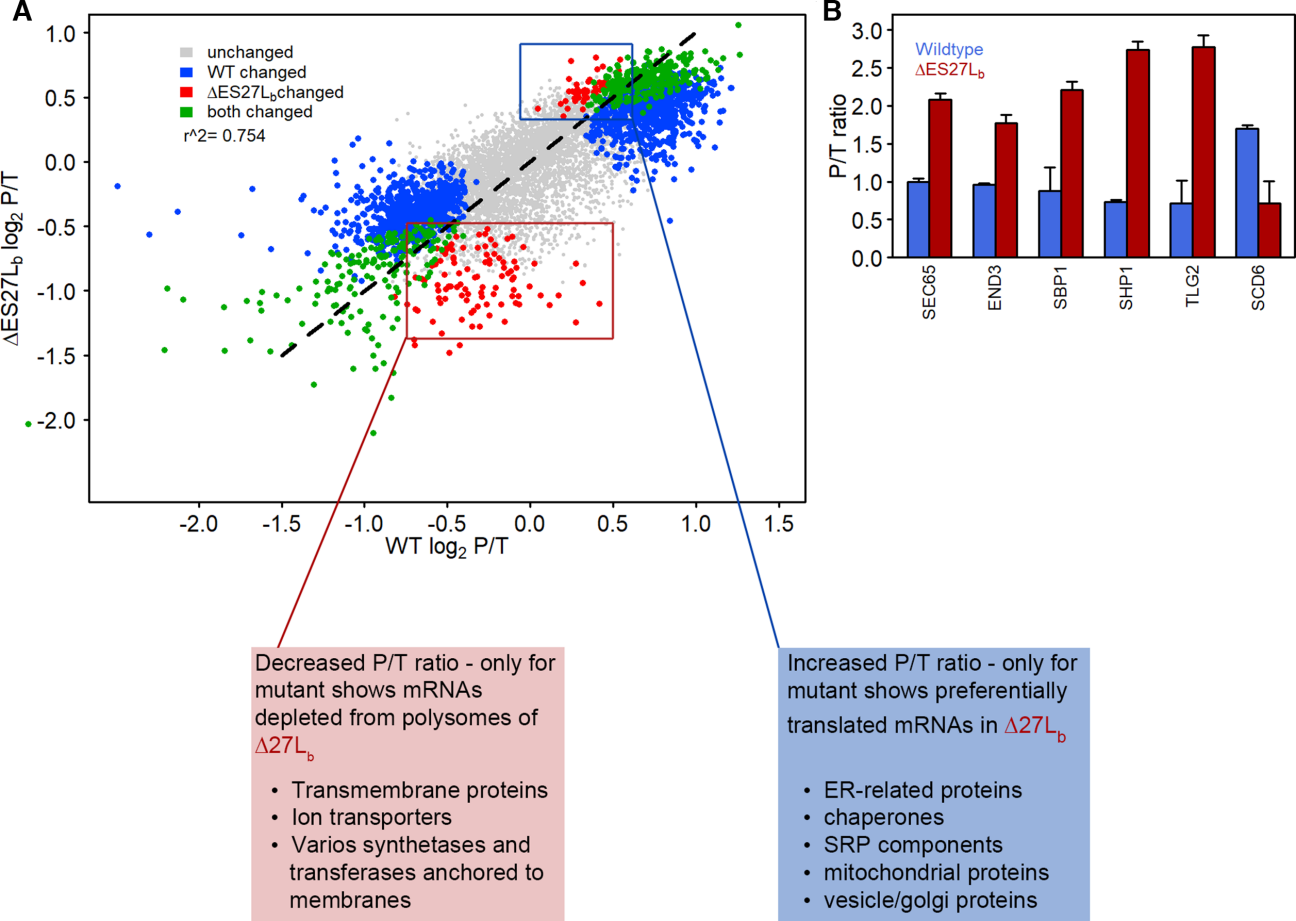
Polysome mRNA-Seq analysis. (**A**) Scatter plot showing mRNAs identified in ΔES27Lb (y-axis) and wildtype (x-axis) cells. The amount of ribosome-association of mRNAs was calculated as the ratio of polysome-bound mRNA reads over total mRNA reads (P/T ratio) and plotted as log_2_-P/T ratio. Data were analyzed with DESeq2 for significantly altered translation (*P* ≤ 0.05) of mRNA transcripts for either the ΔES27Lb mutant strain alone (red dots), or the wildtype strain alone (blue dots) or as a combination in both strains (green dots). Boxes highlight groups of mRNAs with different P/T ratio in ΔES27Lb. The red dots in the lower left quadrant of the plot represent mRNAs that are decreased in polysomes of ΔES27Lb cells (red box), while the red dots on the upper right corner comprise mRNAs that are enriched in polysomal fractions in the ΔES27Lb cells (blue box). Textboxes give short descriptions of protein functions encoded by these differentially translated mRNAs. (**B**) Verification of P/T ratios for candidate mRNA transcripts by RT-qPCR. *SEC65, END3, SBP1, SHP1* and *TLG2* are, based on polysome mRNA-Seq, examples of mRNAs proposed to be preferentially translated by ΔES27Lb ribosomes while *SCD6* was from the list of underrepresented polysomal mRNAs in the mutant cells. Data shown represent the mean and SD of three independent biological replicates.

To determine whether the ΔES27Lb ribosomes translated specific sets of mRNAs, polysomal mRNA sequencing was carried out in triplicates. mRNA-Seq was performed for poly(A)-containing RNA isolated from total cell lysates and also for mRNA isolated from the polysomal fractions of polysome profiles. The translational efficiency was calculated and expressed as a log_2_ ratio between polysome-associated mRNA reads (P) and total mRNA reads (T). A comparison of the log2-fold changes of the translation efficiency between wildtype and ΔES27Lb ribosomes shows only minor differences between the two strains (Figure 3A). Interestingly, DESeq analysis revealed a small and highly specific pool of mRNAs to be differentially translated by the ΔES27Lb ribosomes (Figure 3A, red dots; Supplementary Tables S3 and S4). The list of mRNAs that are more frequently translated in the case of the mutant include many that encode components of ER membrane, components of ER-to-Golgi transport vesicles and the translocon, chaperones of both the cytoplasm and the mitochondria and a few mitochondrial ribosomal proteins (Figure 3A). Transcripts that are under-represented in the actively translating pool of ribosomes encode mostly transmembrane proteins of different categories—ER, mitochondria, Golgi, plasma membrane and other vesicles (Figure 3A). At the same time, transcriptome comparison between the wildtype and the ΔES27Lb cells did not show significant differences (Supplementary Figure S5) thus pointing to alterations at the translational rather than transcriptional level. These sets of differentially translated messages hint toward disturbed protein folding, targeting and other post- or co-translational protein quality control mechanisms. Specifically, there appears to be less translation of mRNAs coding for membrane proteins or proteins intended for localization within membranes of organelles, and concurrently, enhanced translation of mRNAs coding for certain chaperones (required for reducing the amounts of misfolded proteins), translocon and SRP complex (required for proper protein targeting). qRT-PCR for exemplary differentially translated mRNAs confirmed the observations of the RNA-Seq analyses (Figure 3B). Thus, deletion of the b-arm of ES27L, while not affecting the phenotype during optimal growth conditions, altered the translatome of these mutant cells slightly but specifically.

### Deletion of ES27Lb causes increased cellular protein aggregation

**Figure 4.**
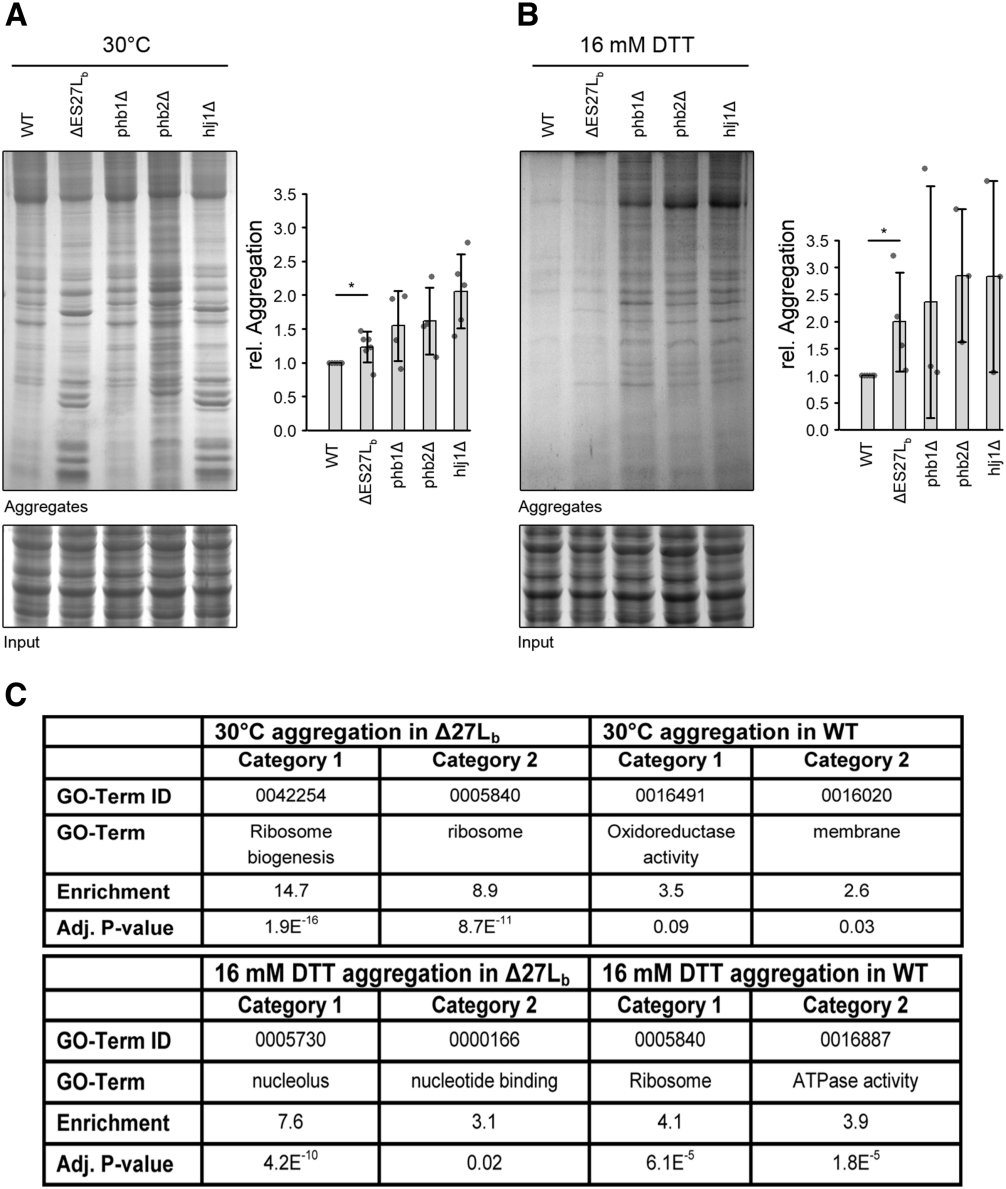
Protein aggregation and the influence of chaperones. (**A**) Representative SDS polyacrylamide gel of aggregated proteins derived from wildtype cells (wt), from cells lacking ES27Lb or from cells lacking either of the three chaperones phb1Δ, phb2Δ or hlj1Δ. Cells were grown under normal conditions (30°C) (**A**), or stressed for 1 h at 16 mM DTT (**B)**. Coomassie-stained proteins from total lysates serve as loading control (input). Diagrams indicate quantifications of aggregates normalized to wt in each condition. Data are mean ± SD. (**C**) GO-Terms with highest enrichment score according to DAVID analysis for the respective conditions. Specifically aggregated proteins were defined as those proteins appearing uniquely in one dataset or which were more than two-fold enriched in the respective condition.

It is known that perturbed protein quality control pathways result in misfolded proteins forming aggregates in the cell. Chaperones and the proteasome assist in maintaining low levels of aggregated proteins preventing them from becoming toxic to the cells (33). Given that mRNAs coding for chaperones (*PHB1, PHB2* and *HLJ1*) are more efficiently translated in the ΔES27Lb strain and coupled with the observation of increased sensitivity toward reductive stress, it is possible that proteins are more prone to aggregation in the ΔES27Lb cells. Therefore the aggregation status was assessed by isolating aggregates by repeated rounds of sonication of cell lysates (18) obtained from cells grown under optimal conditions, or during DTT stress. The insoluble aggregates were isolated by centrifugation and run on denaturing gels. It became apparent that protein aggregation under optimal growth condition was slightly but significantly more pronounced in the case of ΔES27Lb cells than in the case of wildtype (Figure 4A), even though, phenotypically, the cells do not seem to be affected by these aggregation events (Figure 1A, C). However, concurrently taken with the increased translation of chaperones in the ΔES27Lb strain, it would argue for a compensatory mechanism in which this increased translation of certain chaperones assists in managing the elevated protein aggregation events. To test whether elevated chaperone levels in the ΔES27Lb strain counteract deleterious effects on protein folding we created individual chaperone deletion strains in our wildtype background. When proteins were isolated from the three chaperone deletion strains (*phb1Δ, phb2Δ* and *hlj1Δ*) grown under normal conditions, an increased aggregation propensity was detected (Figure 4A). These results mirror the situation in the ΔES27Lb strain and support the hypothesis, that these chaperones are upregulated (Figure 3A and Supplementary Tables S3 and S4) to counteract protein misfolding. When we exposed cells to DTT, the extent of aggregates in the case of the ES27Lb strain is elevated compared to the wildtype control strain (Figure 4A, B). This provides a possible explanation for the slight growth defect observed under reductive stress conditions (Figure 1A). Perhaps these specific aggregates are formed as a consequence of misregulated protein quality control. Treating the chaperone knockout strains with DTT caused enhanced protein aggregation as compared to wildtype and ΔES27Lb cells (Figure 4B). Importantly, however, the pattern of protein aggregation observed is very similar in ΔES27Lb and the chaperone knockout strains suggesting a similar subset of proteins to be vulnerable to aggregation in ΔES27Lb and the individual chaperone knockout strains. To obtain further insight into these specific aggregation events, we purified the aggregated proteins and determined their composition via MS. Whereas no proteins were enriched in wildtype cells that were connected to any particular cellular function, aggregates formed in the ΔES27Lb strain were clearly enriched in proteins involved in ribosome biogenesis and in RPs (Figure 4C). These results correlate with the reduced levels of RPs in whole cell lysates (Supplementary Figure S4D). Apparently, RPs are particularly sensitive to aggregation in the ΔES27Lb cells. Facing DTT stress, RPs become aggregation-prone also in wildtype cells, whereas nucleolar proteins, and thus comprising primarily ribosome biogenesis factors, form the majority of aggregated proteins in the ΔES27Lb strain (Figure 4C). Taken together, these results suggest an increased aggregation propensity of RPs and ribosome biogenesis factors in ΔES27Lb cells.

**Figure 5.**
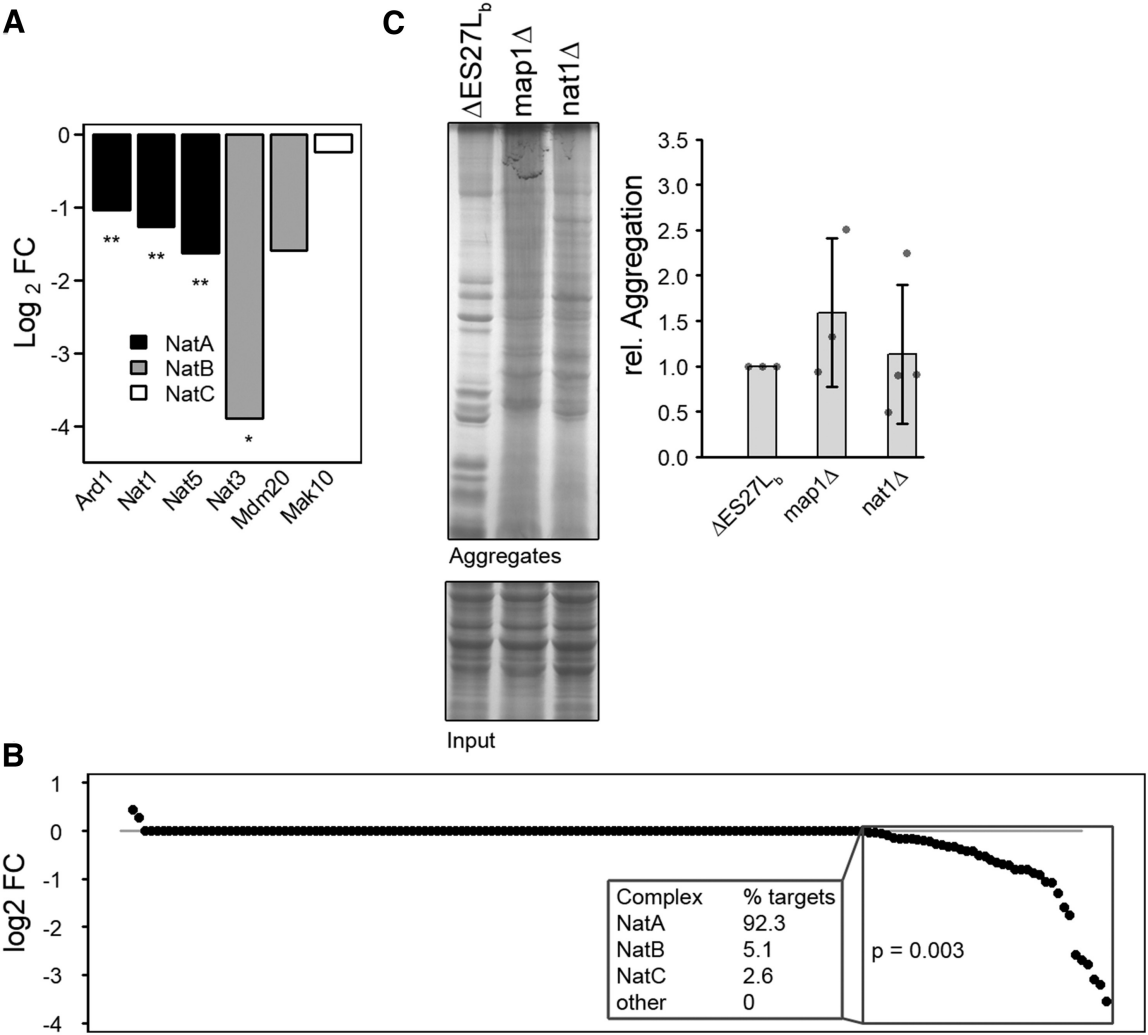
Protein aggregation in *nat1*Δ and *map1*Δ strains. (**A**) Alterations in protein association levels of various components of Nat complexes to the ribosome were assessed by mass spectrometry and quantified by Max Quant. Changes in ribosome-association are shown as log_2_-fold changes. Names of protein constituting the complexes are given below. Asterisks indicate the significance of fold changes (* *P* ≤ 0.05, ** *P* ≤ 0.01). (**B**) Relative levels of acetylated N-termini in ΔES27Lb cells as compared to wildtype cells. All proteins for which N-terminal peptides could be reliably detected in the MS dataset were tested for their acetylation status which was further quantified. Data is shown as log_2_ fold change. Each dot represents a single protein. The *P*-value signifies the significance of the shift of distribution of acetylation frequencies between wildtype and ΔES27Lb. The box highlights the group of proteins with significantly reduced acetylation levels in the ΔES27Lb strain. (**C**) SDS polyacrylamide gel of aggregated proteins isolated from the ΔES27Lb strain or from cells chromosomally lacking Map1 (*map1*Δ) or Nat1 (*nat1*Δ). Coomassie-stained proteins from total lysates serve as loading control (input). Bar graph on the right shows quantifications of aggregates normalized to ΔES27Lb. Data are mean ± SD.

Earlier studies correlated the acetylation status of proteins with their aggregation propensity (34,35). Misacetylation has been shown to either, and depending on the protein identity, increase or decrease aggregation (26). As we had observed reduced ribosome-association of the NatA and NatB complexes (Figure 5A), as well as Map1, an N-terminal methionine peptidase (Supplementary Table S2), we envisioned reduced N-terminal acetylation and consequently an altered aggregation tendency of proteins in the ΔES27Lb strain. To test this hypothesis, we first compared N-terminal fragments within our MS data of 163 proteins which were detected in all samples and which showed unambiguous signs of acetylation. Indeed, for these proteins we found a significant trend toward reduced acetylation frequencies as compared to wildtype (Figure 5B). Next, we asked whether proteins that aggregated display distinct dependencies on Nat-complexes. To this end, we stratified the proteins exclusively found in the aggregates of wildtype or ΔES27Lb cells according to their N-terminal amino acid which defines the Nat complex substrate specificity (26). While there were no striking differences in Nat complex specificity of aggregated proteins in the wildtype or ΔES27Lb cells (Supplementary Figure S6), a slight tendency became apparent toward NatA acetylation targets in the aggregates. This is in agreement with NatA’s significant absence from polysomes (Figure 5A) and the specific reduction of acetylation on NatA protein targets (Figure 5B). Finally, to learn whether the complete absence of Map1 or the NatA complex from the ribosome increases the aggregation tendency, we created *map1* and *nat1* knockout strains in our wildtype background and subsequently performed aggregation assays. In case of*map1*Δ cells, we observed a slight increase in aggregation tendency (Figure 5C) whereas the increase of aggregated proteins in *nat1*Δ cells followed that trend, yet did not reach significant levels. Taken together, these results suggest a causal link between hampered Map- and Nat-mediated N-terminal protein modifications and protein aggregation. Thus, perturbed ribosome association of specific Nat-complexes due to the absence of ES27Lb alters the specific acetylation patterns and consequently renders a specific subset of proteins aggregation prone.

## DISCUSSION

Even though eukaryal ribosomal RNA expansion segments (ES) have been known for decades, dedicated studies aiming at unraveling their contribution to ribosome biology and ribosome biogenesis have only emerged recently (5,7,10,11). Despite the employment of state-of-the art biochemical, structural and cell biological approaches, the physiological role(s) of ES are still poorly understood in molecular terms. ES27L is the second largest expansion segment in the yeast 25S rRNA, with its base originating from helix 63 of the rRNA (Supplementary Figure S1) (36). As one of the most dynamic expansion segments, ES27L was not readily resolved in high resolution structures, but modeling places two conformations for the ES27Lb arm—one close to the L1 stalk and one toward the nascent peptide exit tunnel (28). Sweeney *et al.* described that ES27L is essential in Tetrahymena, as deletion of ES27L from the ribosome resulted in lethality (27). However, the phenotype was rescued by replacing the deleted arm with sequences of ES27L from other organisms, but not random sequences. While the sequences of ES27L share very little sequence identity between species, there is a structural similarity, arguing for a structural function of ES27L on the ribosome (27).

In this study, we characterize the role of yeast ES27Lb of the large ribosomal subunit. In particular we focused on the b-arm of ES27 located at helix 63 in domain IV of 25S rRNA in the yeast 80S ribosome in protein synthesis and describe the wide-ranging effects of the loss of this rRNA arm for ribosome functioning, the yeast proteome and cell viability. Deletion of the entire expansion segment ES27L was lethal, as also observed by others (7,27). However, upon removal of ∼90 nucleotides from the b-arm (Figure 1B, Supplementary Figure S1), cells were viable and showed no growth phenotype in normal medium but revealed a slightly reduced biological fitness during reductive stress (Figure 1A, Supplementary Figure S2). Furthermore, we observe a slightly changed subunit ratio in polysome gradients (Figure 2A), indicative of potential deficits at the level of large ribosomal subunit biogenesis. In steady state, however, total levels of mature rRNA and polysomes are unaltered (Supplementary Figure S3 and Figure 2A, B) strongly indicating the availability of sufficient amounts of translationally-competent ribosomes in the ΔES27Lb strain. This is in clear contrast to other previously described rRNA ES deletions that resulted in dramatic ribosome biogenesis defects and consequently strongly impaired growth (10). Additionally, we also observed a decrease in the levels of ribosomal proteins of the large subunit (RPLs) in whole cell lysates (Supplementary Figure S4D), indicating an excess of free RP over rRNA in the ΔES27Lb cells. Perhaps the lack of ES27Lb affects the kinetics of 60S subunit maturation, and these premature particles are actively cleared away, offering a possible explanation for the reduced free 60S subunit levels in polysome profiles (Figure 2A, B). The unincorporated RP accumulate in the cell and are likely the driving force for activated proteome homeostasis pathways, protein clearance and cellular aggregation. Similar effects have been observed for aberrantly synthesized ribosomes in the absence of rRNA processing factors (37). Unexpectedly, we observed 20S precursor accumulation which gives rise to the 18S rRNA and whose maturation is decoupled from 25S rRNA processing. This finding suggests that ribosome biogenesis *per se* is slightly perturbed in the ΔES27Lb strain. The fact that ribosome biogenesis factors significantly aggregate in the ΔES27Lb strain (Figure 4C) can explain this overall decrease in ribosome biogenesis efficiency. However, we observe that notwithstanding a mild biogenesis defect, ΔES27Lb large subunits are correctly processed and engage in active translation (Figure 2). While protein synthesis *per se* and 60S subunit functioning in particular does not seem to depend on the presence of the b-arm of ES27L, its absence influences the ribosome interactome and as a consequence might alter the proteome. Indeed, by assessing the translation efficiency index of each mRNA it became apparent that removal of the ES27L b-arm affects the mRNA association to the ribosomes and thus shapes the proteome (Figure 3). In ΔES27Lb mutant cells we observed an increased translation of mRNAs encoding for specific chaperones, ER proteins, and mitochondrial chaperons that can assist in protein folding and targeting of nascent chains, coupled with a concomitant decrease in translation of mRNAs coding for various membrane proteins (ER, Golgi, plasma membrane and vesicles) (Figure 3). We hypothesize that this altered translatome is a consequence of changed association of certain ribosome associated protein biogenesis factors (RPBs; such as Map and Nat), and the increased translation of specific chaperone mRNAs (*PHB1, PHB2* and *HLJ1*) serves to compensate for increased aggregation propensity. Our experimental data support this scenario since cells lacking ES27Lb indeed show an increased protein aggregation propensity primarily involving RPs and ribosome biogenesis factors (Figure 4). Moreover, the pattern of aggregation observed in ΔES27Lb cells is pheno-copied in chaperone knockout cells suggesting that these chaperones indeed are upregulated to counteract ΔES27Lb induced protein aggregation (Figure 4A). In particular the aggregation pattern observed in the *hlj1* deletion strain, an HSP40 co-chaperone in the ER, is indistinguishable from the ΔES27Lb strain. From a mechanistic point of view we propose that loss of Map and Nat from the ΔES27Lb ribosomes result in nascent peptides lacking the necessary modifications (methionine removal/acetylation) that are required for the proper functioning of proteins. Both Map and Nat are the first co-translational protein modifiers a nascent chain encounters and that are required for further downstream processing (reviewed in 38). In the case of ΔES27Lb ribosomes, lack of complete N-terminal modifications renders proteins aggregation prone (especially during stress). To compensate for this challenging situation it is possible that cells react by avoiding synthesizing proteins heavily depending on N-terminal modifications and by up-regulating certain chaperones coping with the aggregates, thus altering the proteome.

It has been speculated that ribosomal ES evolved primarily as binding platforms for recruiting additional proteins to the translation machinery in order to add another layer of translation control. To test this hypothesis and to gain global insight into the proteins associated with translating ribosomes that lack ES27Lb, MS–based analysis of the polysome-associated proteome has been conducted. Our results demonstrate proteins of the methionine amino peptidase Map1/2 and the N-terminal acetylation complex NatA and NatB to be significantly less abundant in the ΔES27Lb polysomes (Supplementary Table S2). These findings are in line with very recent findings of two other groups (5,7) therefore validating our MS-based approach. Together these findings strongly suggests that ES27Lb is indeed the prime ribosomal attachment element for these ribosome-bound enzyme complexes involved in modifying the N-termini of emerging polypeptides in a co-translational manner. During the final stages of this study, Fujii *et al.* published a paper describing results of a very similar ES27L deletion strain (7). Our observation of the methionine amino peptidase Map1/2 being underrepresented on the ΔES27Lb ribosomes is in agreement with the conclusion made by Fujii and colleagues. While in the latter publication the authors primarily focused on differences on the composition of ribosome-associated proteins between the wildtype and ΔES27Lb ribosomes, our study embraces also other aspects of ribosome biology such as comparison of the translated mRNA pools, effects on proteostasis and its consequences on cellular fitness upon stress. For example, our protein aggregation experiments, reveal insight that goes beyond the mere description of the ribosomal binding site for the methionine amino peptidase Map1/2 and the N-terminal acetylation complex NatA and NatB. We could demonstrate that proteins that are targeted for N-terminal acetylation by the NatA and NatB complex, showed indeed decreased levels of acetylation in cells lacking ES27Lb and concomitantly increased predisposition for aggregation upon reductive stress stimuli (Figure 4). While Fujii *et al.* (7) suggested mistranslation of ribosomal proteins in the ΔES27Lb strain causing error prone ribosomes, we demonstrate an increased tendency of ribosomal proteins for aggregation that could thus contribute to the phenotype described in the above mentioned publication.

In previous studies, stress-dependent compromised protein folding led to adapted cellular translation of specific chaperones and RPBs in order to re-establish protein homeostasis (33,39). Corollary to that, deletion of the ES27Lb arm on the ribosome might be one such event where the truncated ribosomes have adjusted their translation specificity to compensate for the protein folding stress. While that might be sufficient to maintain the cellular protein aggregation at a level that does not perturb normal growth (Figure 1A, C), during reductive stress, however, the proteostasis can no longer be maintained. Typically misfolded proteins and aggregates are either re-solubilized by the help of dedicated chaperones (40) or handed over to the proteasome for degradation (41–43). In the case of cells depending on ΔES27Lb ribosomes for synthesizing their proteomes, the well-adjusted cellular network that typically copes with misfolded and/or aggregated proteins gets out of balance. As a consequence, increased protein aggregation is observed (Figure 4) which results in an increased sensitivity of the mutant cells for reductive stress thus explaining the growth defect evident in the presence of DTT (Figure 1A). In another recent report data have been presented that also involved a yeast 25S rRNA ES in redox stress response, namely ES7c (32). The authors observed an endonucleolytic cleavage within ES7c upon mild DTT stress that likely modulates the ribosomes’ performance during early stages of stress adaptation. Cumulatively, the picture that emerged in these recent studies on ES is compatible with the view that these extra rRNA elements enable the translation machinery to fine-tune its activity and specificity under emerging challenging conditions or during the need for elaborated protein targeting in eukaryal cells. This in turn might explain the driving force for expanding the ribosome at the rRNA and the RBP levels during the course of evolution from single-cell prokarya to more sophisticated multicellular eukarya.

## DATA AVAILABILITY

mRNA-Seq sequencing data have been deposited at GEO database under the accession number GSE134774.

https://www.ncbi.nlm.nih.gov/geo/query/acc.cgi?acc=GSE134774

## Supplementary Material

gkaa003_Supplemental_File

## Notes

Present address: Vaishnavi Shankar, Department of Microbiology and Molecular Medicine, University of Pittsburgh, 543 Bridgeside Point II 450 Technology Dr. Pittsburgh, PA 15219, USA.
