# Supplementary material for: rRNA expansion segment 27Lb modulates the factor recruitment capacity of the yeast ribosome and shapes the proteome
Source: Nucleic Acids Res. 2020 Jan 21;48(6):3244–56. doi: 10.1093/nar/gkaa003 (PMC7102955; doi:10.1093/nar/gkaa003)
Supplement: gkaa003_Supplemental_File [file gkaa003_supplemental_file.pdf]

Supplementary Data

rRNA expansion segment 27Lb modulates the factor recruitment capacity of the yeast ribosome and shapes the proteome

Vaishnavi Shankar[1,2,#,†], Robert Rauscher[1,†], Julia Reuther[1], Walid H. Gharib[3], Miriam Koch[1] and Norbert Polacek[1*]

[1]Department of Chemistry and Biochemistry, University of Bern, Freiestrasse 3, 3012 Bern, Switzerland

[2]Graduate School for Cellular and Biomedical Sciences, University of Bern, Bern, Switzerland

[3]Interfaculty Bioinformatics Unit, University of Bern, Baltzerstrasse 6, 3012 Bern, Switzerland

#current address: Department of Microbiology and Molecular Medicine, University of Pittsburgh, 543 Bridgeside Point II 450 Technology Dr. Pittsburgh, PA 15219, USA

[†]The authors wish it to be known that, in their opinion, these two authors should be regarded as Joint First Authors.

*To whom correspondence should be addressed:

norbert.polacek@dcb.unibe.ch

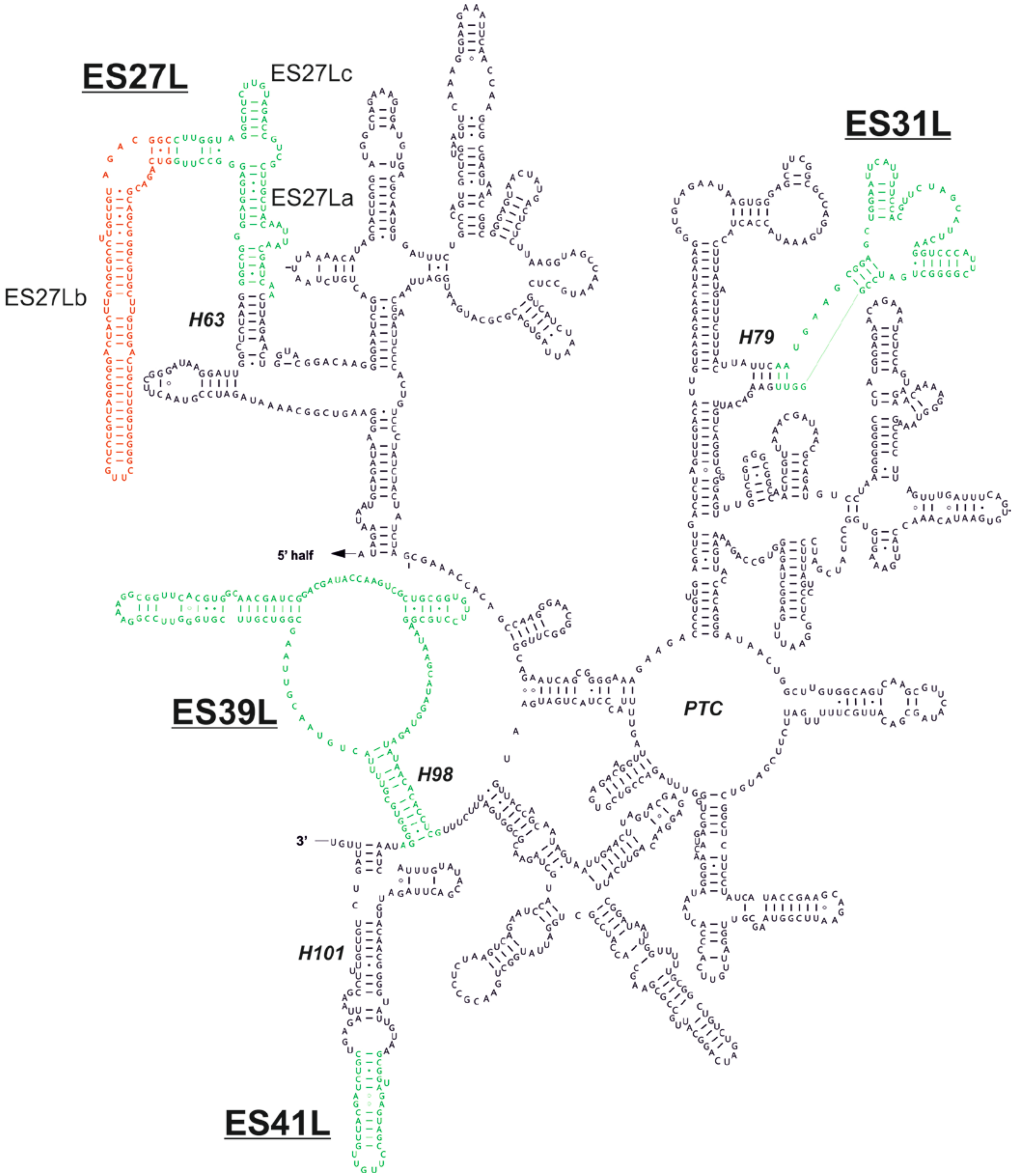

Supplementary Figure S1: Secondary structure of the 3'-half of S. cerevisiae 25S rRNA. The four ES in the 3'-half of 25S rRNA are labeled in green and the corresponding rRNA helix is indicated. The b-arm of ES27L, which was deleted in this study, is highlighted in red. PTC denotes the location of the peptidyl transferase center.

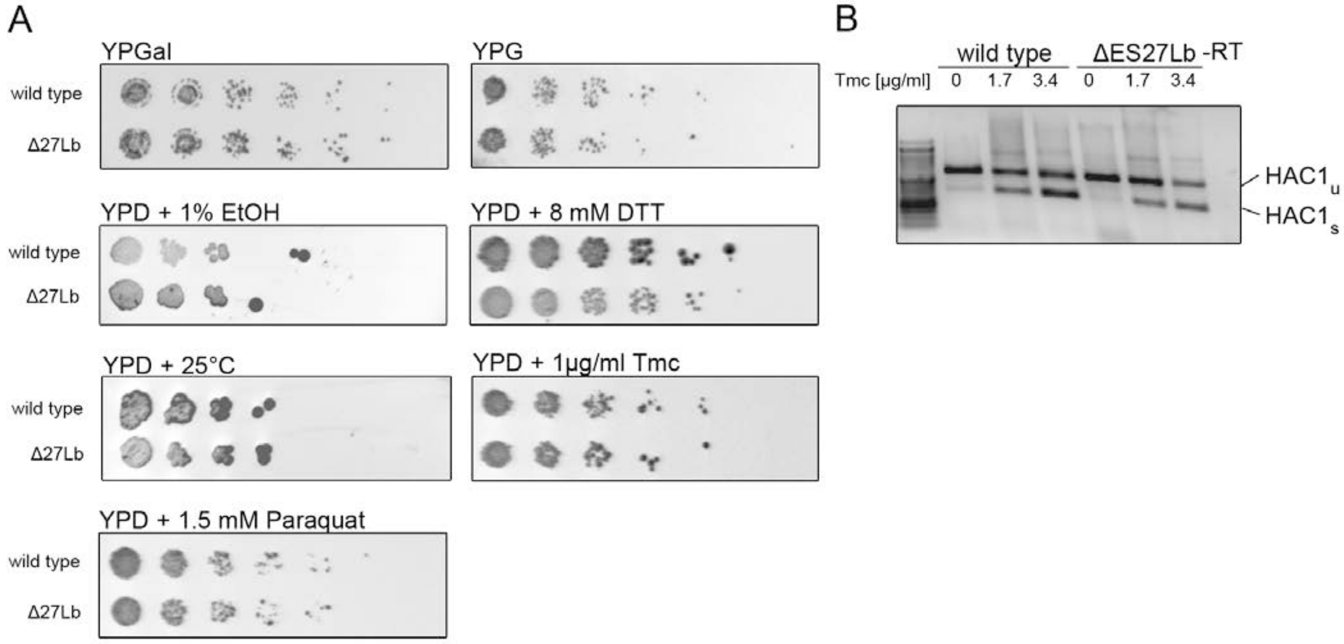

Supplementary Figure S2: Intrinsic ER stress response in the ΔES27Lb strain. (**A**) Cells were diluted to OD_{600} = 0.1 and spotted (serial dilutions of 1/10) on to agar plates containing the indicated compounds. If not stated otherwise plates were incubated at 30°C. (**B**) Wildtype and Δ27Lb yeast were incubated with increasing amounts of the ER-stress inducing drug tunicamycin (Tmc). HAC1 splicing was assayed by RT-PCR with primers amplifying the spliced region. -RT indicates a negative control without reverse transcription. No difference in the ration between unspliced ($HAC1_u$) and spliced ($HAC1_s$) *HAC1* mRNA was observed between wildtype and ΔES27Lb cells.

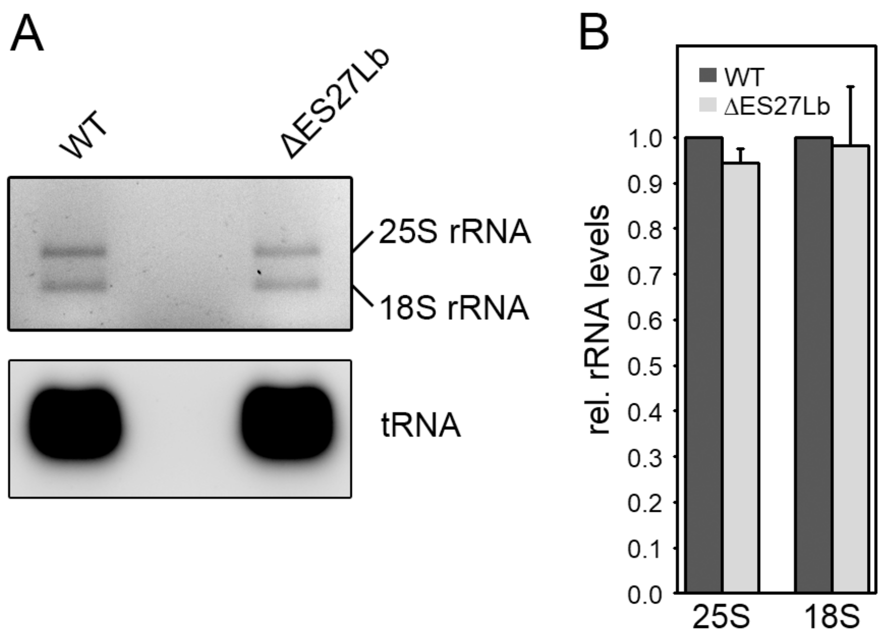

Supplementary Figure S3: Levels of mature rRNAs in the wildtype and ΔES27Lb strain. (**A**) Ethidium bromide stained gel showing the mature 18S and 15S rRNA in the wildtype (WT) and in the ES27Lb deletion strain (ΔES27Lb). tRNA bands serve as loading control. (**B**) Quantification of 18S and 25S rRNA levels from four biological replicates (mean and standard deviations are shown).

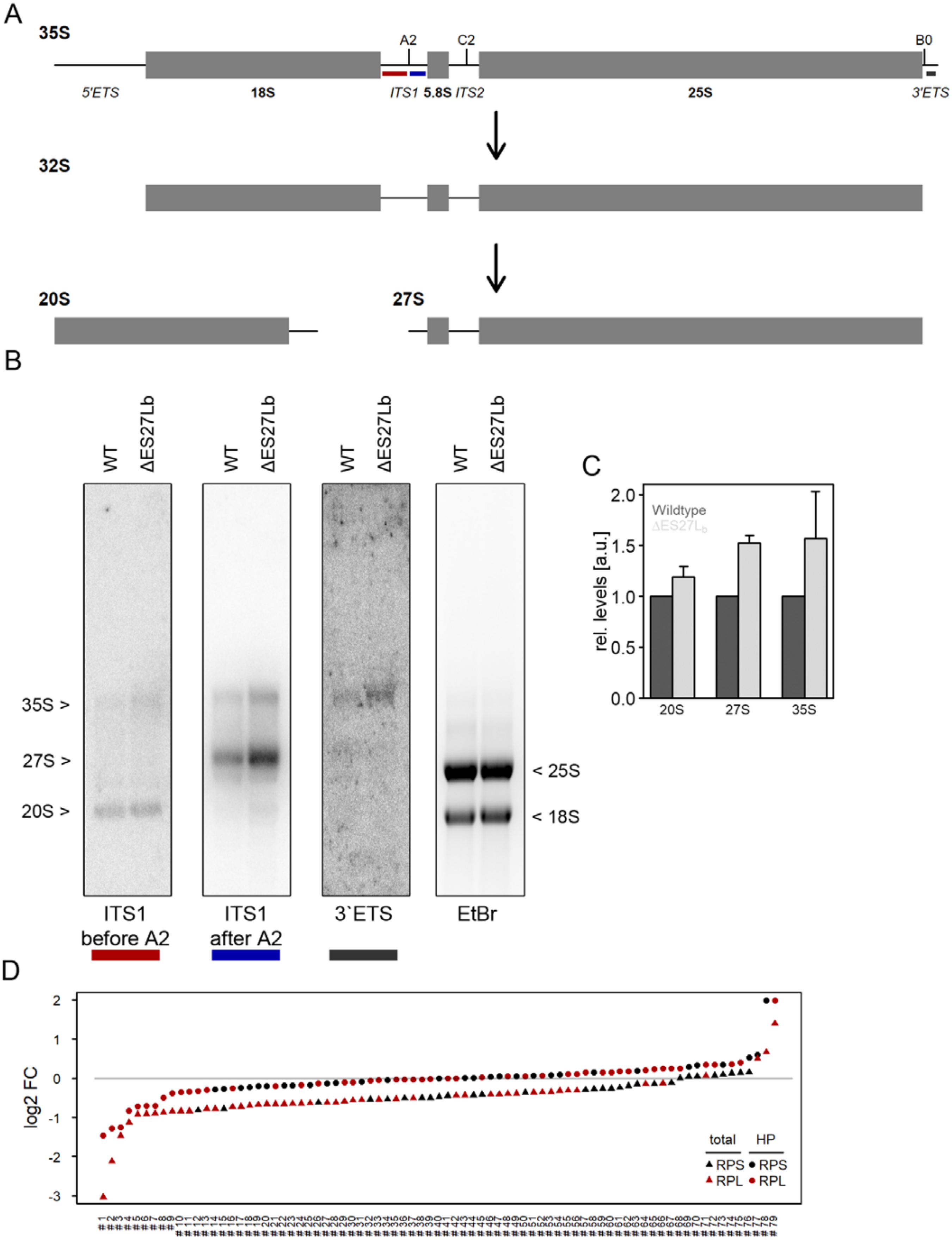

A
35S
A2
C2
B0
5'ETS
18S
ITS1
5.8S
ITS2
25S
3'ETS
32S
20S
27S
B
WT
ΔES27Lb
35S >
27S >
20S >
< 25S
< 18S
ITS1 before A2
ITS1 after A2
3`ETS
EtBr
C
Wildtype
ΔES27Lb
rel. levels [a.u.]
2.0
1.5
1.0
0.5
0.0
20S
27S
35S
D
log2 FC
2
1
0
-1
-2
-3
total
HP
RPS
RPL

Supplementary Figure S4: rRNA maturation and ribosomal protein levels. (**A**) Schematic representation of the rDNA gene. Boxes show rRNA present in mature ribosomes and lines indicate pre-rRNA regions removed during processing (ETS: external transcribed spacer, ITS: internal transcribed spacer). The red, blue and grey bars indicate the positions of the probes used to detect specific processing intermediates. (**B**) Total RNA from wildtype and two Δ27Lb clones separated on denaturing gels and used for northern blot analysis employing the indicated northern blot probes, capable of detecting the presence of 35S, 27S and 20S precursor rRNA transcripts. The ethidium bromide stained gel (right) showing 18S rRNA and 25S rRNA before transfer serves as loading control. (**C**) Quantification of the 20S, 27S and 35S precursor RNA obtained in the DES27Lb strain compared to the wildtype cells (set to 1.0). The mean and standard deviations of three independent experiments is shown. (**D**) Ribosomal proteins (RP) were quantified by mass spectrometry in whole cell lysates (total; triangles)) and in heavy polysomes (HP; dots) after sucrose density centrifugation (active translating ribosomes; red). Ribosomal proteins from the large (RPL) and small (RPS) subunit are depicted in red and black, respectively. Changes of RP abundance were quantified using Max Quant and $\log_2$-fold changes between wildtype and ΔES27Lb cells were plotted. Fold-changes were ordered by their magnitude for each dataset plotted.

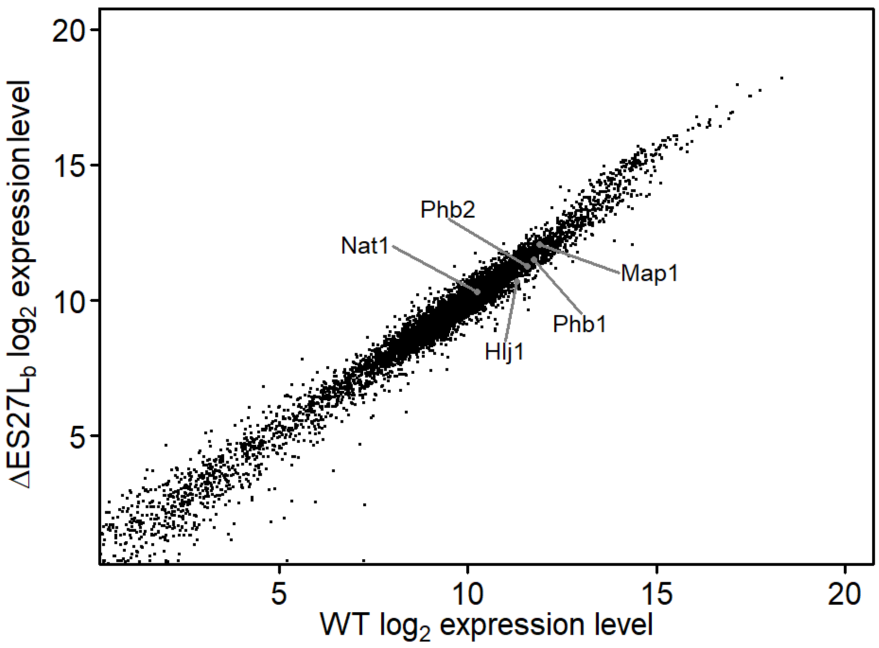

Supplementary Figure S5: Whole mRNA transcriptome comparison. Total mRNA expression was quantified by deep sequencing of cDNA libraries derived from exponentially growing wildtype (WT) or ΔES27Lb cells. Counts were normalized using DESeq with standard parameters and plotted on log2 scale. The highlighted mRNA transcripts depict those genes further investigated in aggregation assays.

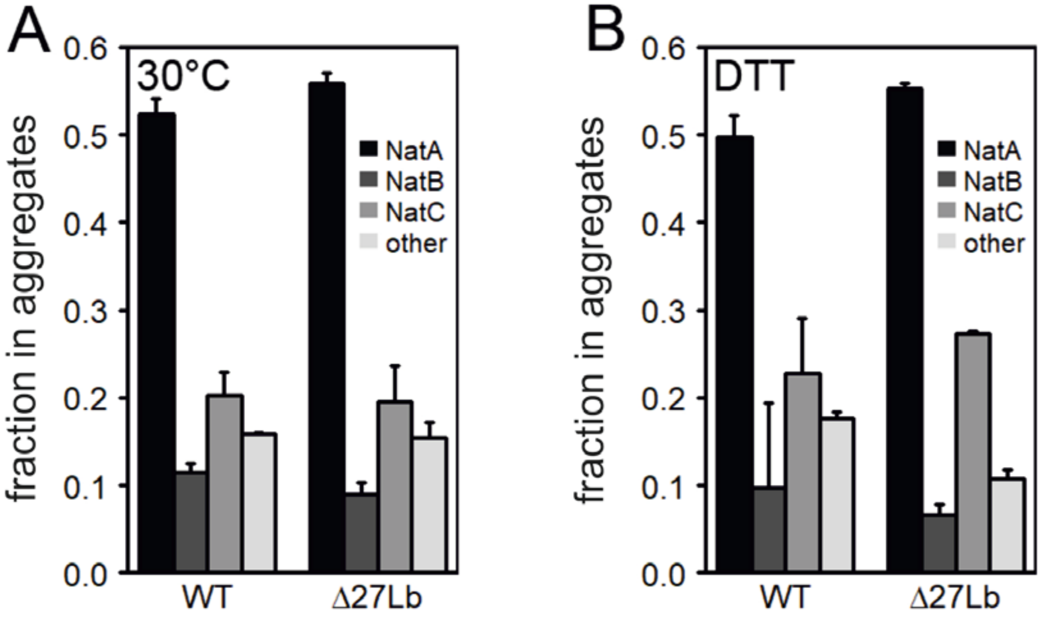

Supplementary Figure S6: Nat-complex target composition of specifically aggregated proteins. Proteins that specifically aggregated in either wildtype (WT) or ΔES27Lb cells were sorted according to their first amino acid and subsequently attributed to the Nat complex with specificity for that amino acid. The ratio for each group over total number of proteins was calculated and is presented as fraction of aggregates. Aggregates derived from cells grown under normal conditions **(A)** or 8 mM DTT stress **(B)** were compared (n = 2, data shown as mean ±SD).

Supplementary Table S1

qRT-PCR primers

S.c.-SBP1 F-2nd	GTCAGCGTAGAGATCCCAATTA
S.c. SBP1 R-2nd	GCCTCTGAAACCTCCTCTAAA
S.c.-END3 F	GTGGCAGTAACGTGCTTTC
S.c.-END3 R	GCTTGCAATTCGTGTCTCTTA
S.c.-SEC65 F	GATAACGCCTAAGGTTGTAAGG
S.c.-SEC65 R	CCCTTCTCCCTTCCTTATGT
S.c.-NUP53 F	GAAGCTAGTGCAGGGAGTAA
S.c.-NUP53 R	TCAACTTGACCCATCCATCT
S.c.-SHP1 F	CTGAACCAACCAAGGGTAGTA
S.c.-SHP1 R	CCATTTCATGGTCTTCGTCATC
S.c.-TLG2 F	GTGCAGAGAACACGCTATTATT
S.c.-TLG2 R	CCCTGGTCAACAACCAAATC
S.c.-SCD6 F	GTTCCTCAAATGATGCCACC
S.c.-SCD6 R	AGCGGTTTCTGTCGAAGTTGG
S.c.-SIP18 F	CGCTGAAAAATTACAAGGTAACG
S.c.-SIP18 R	CGTAAGTCTTCCAATCGTTCG
18S-rRNA F	AATCATCAAAGAGTCCGAAGACATTG
18S-rRNA R	CCTTTACTACATGGTATAACTGTGG
CDC19-F	CAGAGGTGACTTGGGTATTG
CDC19-R	GGTTGGTCTTGGGTTGTAAG
PGK1-F	GGAACACCACCCAAGATAC
PGK1-R	GGTGACATCCTTACCCAAC

ES deletion primers

ES27Lbfwd	GTGAGGGCCCTTGGGTAGGTCTCTTGTAGACCGTCGCTTGC
ES27Lbrev	GACCTACCCAAGGGCCCTCACTACCCGACCCTTAGAGCC

Northern blot probes

ITS2 probe (FL185)	GGCCAGCAATTTCAAGTTA
25S rRNA probe (D)	CTCACCCTCTATGACGTCCTGTTC
ITS1_1 probe	CGGTTTTAATTGTCCTA
ITS1_2 probe	CCAGTTACGAAAATTCTTGTTT
3'ETS probe	TCCTGCCAGTACCCACTT

Morgan analysis primer

S.c._Morgan_ES27Lb	TCTACAAGAGACCTACC

All DNA oligonucleotides used in this study are listed and given in 5' to 3' orientation.

Supplementary Table S2

Whole cell lysate					
**enriched in ΔES27L_b**			**depleted in ΔES27L_b**		
**GO-Term ID**	**GO-Term**	**Adj. p-value**	**GO-Term ID**	**GO-Term**	**Adj. p-value**
0006355	regulation of transcription	0.284	0005773	vacuole	0.982
0010008	endosome membrane	0.975	0071944	cell periphery	0.988
000636	rRNA processing	0.676	0005886	plasma membrane	0.990
**Polysomes**					
**enriched in ΔES27L_b**			**depleted in ΔES27L_b**		
**GO-Term ID**	**GO-Term**	**Adj. p-value**	**GO-Term ID**	**GO-Term**	**Adj. p-value**
0005886	plasma membrane	0.163	0022626	cytosolic ribosome	0.027
0006621	protein retention in ER lumen	0.428	0031415	NatA complex	0.015
0005783	endoplasmic reticulum	0.996	0046872	metal ion binding	0.260

cytosolic ribosome - 0022626

Protein ID	Log$_2$-foldchange	p-value
Nat1	-1.26	7.72E-7
Nat5	-1.62	6.13E-4
Ard1	-1.04	3.53E-5
Map1	-7.40	1.11E-4

Proteins depleted or enriched in whole cell lysate (upper table) or in the polysomes (actively translating ribosomes; middle table) of ΔES27Lb cells were subjected to GO term enrichment analysis using the DAVID Annotation and Clustering tool (1,2). For each dataset, the three most enriched GO-terms and their respective significance are listed. Underlined are those GO-terms which were found to be significantly enriched in the datasets. The GO-term "cytosolic ribosome" was further detailed in the lower table. The proteins comprising the list are detailed with their respective log$_2$-fold change and the significance of that change as calculated from the MS data using the MaxQuant algorithm (3).

Supplementary Table S3

ID	Gene Name	ΔES27Lb log2FC (P/T)	WT log2FC (P/T)	FC ratio (27Lb/WT)	Description
YGR231C	PHB2	0.51	0.4	1.275	Subunit of the prohibitin complex (Phb1p-Phb2p); prohibitin is a 1.2 MDa ring-shaped inner mitochondrial membrane chaperone that stabilizes newly synthesized proteins; determinant of replicative life span; involved in mitochondrial segregation; prohibitin deficiency induces a mitochondrial unfolded protein response (mtUPR)
YGL130W	CEG1	0.41	0.32	1.28125	Guanylyltransferase involved in mRNA 5' capping; subunit of mRNA capping enzyme
YMR315W	YMR315W	0.58	0.45	1.288888889	Protein with NADP(H) oxidoreductase activity;
YGR086C	PIL1	0.615	0.464	1.325431034	Eisosome core component; eisosomes are large immobile cell cortex structures associated with endocytosis; detected in phosphorylated state in mitochondria;
YOL064C	MET22	0.57	0.43	1.325581395	Bisphosphate-3'-nucleotidase; involved in salt tolerance and methionine biogenesis;
YNL100W	MIC27	0.56	0.42	1.333333333	Component of the MICOS complex; MICOS (formerly MINOS or MitOS) is a mitochondrial inner membrane complex that extends into the intermembrane space and has a role in the maintenance of crista junctions, inner membrane architecture, and formation of contact sites to the outer membrane;
YML105C	SEC65	0.71	0.53	1.339622642	Subunit of the signal recognition particle (SRP); involved in protein targeting to the ER
YPR191W	QCR2	0.61	0.45	1.355555556	Subunit 2 of ubiquinol cytochrome-c reductase (Complex III)
YGR165W	MRPS35	0.53	0.39	1.358974359	Mitochondrial ribosomal protein of the small subunit
YER100W	UBC6	0.59	0.43	1.372093023	Ubiquitin-conjugating enzyme involved in ERAD
YBL091C	MAP2	0.61	0.44	1.386363636	Methionine aminopeptidase; catalyzes the cotranslational removal of N-terminal methionine from nascent polypeptides
YIL021W	RPB3	0.56	0.4	1.4	RNA polymerase II third largest subunit B44;
YGL105W	ARC1	0.62	0.44	1.409090909	Protein that binds tRNA and methionyl- and glutamyl-tRNA synthetases; involved in tRNA delivery, stimulating catalysis, and ensuring localization;
YPR107C	YTH1	0.54	0.38	1.421052632	Essential RNA-binding component of cleavage and polyadenylation factor
YOR298C-A	MBF1	0.79	0.54	1.462962963	Transcriptional coactivator
YLR208W	SEC13	0.615	0.42	1.464285714	Structural component of 3 complexes; subunit of the Nup84p nuclear pore subcomplex that contributes to nucleocytoplasmic transport and NPC biogenesis; subunit of the COPII vesicle coat required for ER-to-Golgi transport; subunit of SEACAT, a subcomplex of the coatomer-related, vacuolar-associated SEA complex, that inhibits the TORC1 inhibitory role of SEACIT (Iml1p-Npr2p-Npr3p), a GAP for Gtr1p, thereby resulting in activation of TORC1 signaling;
YJL167W	ERG20	0.47	0.32	1.46875	Farnesyl pyrophosphate synthetase
YOR111W	YOR111W	0.49	0.33	1.484848485	Unknown
YOR194C	TOA1	0.58	0.39	1.487179487	TFIIA large subunit
YMR161W	HLJ1	0.5	0.33	1.515151515	Co-chaperone for Hsp40p; anchored in the ER membrane
YDL097C	RPN6	0.61	0.39	1.564102564	Essential, non-ATPase regulatory subunit of the 26S proteasome lid; required for the assembly and activity of the 26S proteasome
YGL048C	RPT6	0.52	0.33	1.575757576	ATPase of the 19S regulatory particle of the 26S proteasome
YKL059C	MPE1	0.54	0.34	1.588235294	Essential conserved subunit of CPF cleavage and polyadenylation factor
YPL118W	MRP51	0.55	0.34	1.617647059	Mitochondrial ribosomal protein of the small subunit
YER027C	GAL83	0.74	0.44	1.681818182	One of three possible beta-subunits of the Snf1 kinase complex
YKR055W	RHO4	0.56	0.33	1.696969697	Non-essential small GTPase; member of the Rho/Rac subfamily of Ras-like proteins
YGL001C	ERG26	0.5	0.29	1.724137931	C-3 sterol dehydrogenase; catalyzes the second of three steps required to remove two C-4 methyl groups from an intermediate in ergosterol biosynthesis
YLR418C	CDC73	0.47	0.27	1.740740741	Component of the Paf1p complex; binds to and modulates the activity of RNA polymerases I and II
YGR132C	PHB1	0.54	0.31	1.741935484	Subunit of the prohibitin complex (Phb1p-Phb2p)
YJL081C	ARP4	0.35	0.2	1.75	Nuclear actin-related protein involved in chromatin remodeling; component of chromatin-remodeling enzyme complexes
YOR042W	CUE5	0.62	0.35	1.771428571	Ubiquitin-binding protein; functions as ubiquitin-Atg8p adaptor in ubiquitin-dependent autophagy
YOR158W	PET123	0.72	0.405	1.777777778	Mitochondrial ribosomal protein of the small subunit
YNL177C	MRPL22	0.63	0.34	1.852941176	Mitochondrial ribosomal protein of the large subunit
YGR178C	PBP1	0.41	0.22	1.863636364	Component of glucose deprivation induced stress granules; involved in P-body-dependent granule assembly
YDR322W	MRPL35	0.49	0.26	1.884615385	Mitochondrial ribosomal protein of the large subunit
YNL084C	END3	0.53	0.28	1.892857143	EH domain-containing protein involved in endocytosis
YJR065C	ARP3	0.53	0.27	1.962962963	Essential component of the Arp2/3 complex; Arp2/3 is a highly conserved actin nucleation center required for the motility and integrity of actin patches
YDL236W	PHO13	0.56	0.28	2	Conserved phosphatase acting as a metabolite repair enzyme
YNL173C	MDG1	0.81	0.4	2.025	Plasma membrane protein; involved in G-protein mediated pheromone signaling pathway
YLR079W	SIC1	0.6	0.28	2.142857143	Cyclin-dependent kinase inhibitor (CKI); inhibitor of Cdc28-Clb kinase complexes that controls G1/S phase transition
YPL173W	MRPL40	0.54	0.25	2.16	Mitochondrial ribosomal protein of the large subunit
YBL025W	RRN10	0.47	0.21	2.238095238	Protein involved in promoting high level transcription of rDNA
YGL082W	YGL082W	0.55	0.23	2.391304348	Unknown
YMR153W	NUP53	0.45	0.18	2.5	FG-nucleoporin component of central core of nuclear pore complex (NPC)
YBL058W	SHP1	0.64	0.23	2.782608696	UBX domain-containing substrate adaptor for Cdc48p; ubiquitin regulatory X domain-containing protein that acts as a substrate recruiting cofactor for Cdc48p
YHL034C	SBP1	0.78	0.24	3.25	Protein that binds eIF4G and has a role in repression of translation; has an RGG motif; found in cytoplasmic P bodies; binds to mRNAs under glucose starvation stress, most often in the 5' UTR
YOL018C	TLG2	0.41	0.05	8.2	Syntaxin-like t-SNARE; forms a complex with Tlg1p and Vti1p and mediates fusion of endosome-derived vesicles with the late Golgi

Supplementary Table S4

Gene ID	Name	27LbP/T log2fold	WT P/T log2fold	FC Ratio (27Lb/WT)	Description
YKR102W	FLO10	-0.6752542	-0.5637328	1.197826791	Member of the FLO family of cell wall flocculation proteins; not expressed in most lab strains
YHR186C	KOG1	-0.5883861	-0.448352	1.312330796	Subunit of TORC1; TORC1 is a rapamycin-sensitive complex involved in growth control
YBR295W	PCA1	-0.6911776	-0.5110215	1.352541076	Cadmium transporting P-type ATPase
YLR247C	IRC20	-0.7191879	-0.5123487	1.403707914	E3 ubiquitin ligase and putative helicase
YKL217W	JEN1	-0.7680117	-0.5415597	1.41814765	Monocarboxylate/proton symporter of the plasma membrane; transport activity is dependent on the pH gradient across the membrane
YMR185W	RTP1	-0.6870615	-0.4714764	1.457255313	Protein required for the nuclear import and biogenesis of RNA pol II
YIL013C	PDR11	-0.7443784	-0.4647515	1.601669698	ATP-binding cassette (ABC) transporter; multidrug transporter involved in multiple drug resistance
YGR233C	PHO81	-0.7564424	-0.4627779	1.63456879	Cyclin-dependent kinase (CDK) inhibitor
YJR152W	DAL5	-0.6697863	-0.4067318	1.646751833	Allantoate permease; ureidosuccinate permease
YJL129C	TRK1	-0.5700405	-0.3086935	1.846623047	Component of the Trk1p-Trk2p potassium transport system
YDR459C	PFA5	-0.5263712	-0.2616384	2.011826804	Palmitoyltransferase with autoacylation activity; likely functions in pathway(s) outside Ras
YDL210W	UGA4	-0.8665343	-0.4299744	2.015316142	GABA (gamma-aminobutyrate) permease; serves as a GABA transport protein; localized to the vacuolar membrane
YOR129C	AFI1	-0.5632279	-0.271589	2.073824632	Arf3p polarization-specific docking factor; participates in polarity development and maintenance of a normal haploid budding pattern
YPL258C	THI21	-0.6729541	-0.3172359	2.121305175	Hydroxymethylpyrimidine (HMP) and HMP-phosphate kinase; involved in thiamine biosynthesis
YKR061W	KTR2	-0.829721	-0.3800266	2.183323598	Mannosyltransferase involved in N-linked protein glycosylation
YDR039C	ENA2	-0.6098759	-0.2407476	2.533258098	P-type ATPase sodium pump; involved in Na+ efflux to allow salt tolerance
YAL054C	ACS1	-0.7246089	-0.2628965	2.756251496	Acetyl-coA synthetase isoform; along with Acs2p, acetyl-coA synthetase isoform is the nuclear source of acetyl-coA for histone acetylation
YNL259C	ATX1	-0.7906581	0.27344691	2.891450136	Cytosolic copper metallochaperone; transports copper to the secretory vesicle copper transporter Ccc2p for eventual insertion into Fet3p
YDR545W	YRF1-1	-0.7917505	-0.246977	3.20576616	Helicase encoded by the Y' element of subtelomeric regions
YDL232W	OST4	-1.2722695	-0.3053878	4.166078886	Subunit of the oligosaccharyltransferase complex of the ER lumen
YGL126W	SCS3	-0.6014253	-0.1377783	4.365165998	Protein required for inositol prototrophy; required for normal ER membrane biosynthesis;
YKR097W	PCK1	-0.780831	-0.1642859	4.752880005	Phosphoenolpyruvate carboxykinase; key enzyme in gluconeogenesis
YIL040W	APQ12	-0.588026	-0.1076528	5.462243074	Nuclear envelope/ER integral membrane protein
YPL096C-A	ERI1	-1.0406434	-0.0736271	14.13397182	Endoplasmic reticulum membrane protein that binds and inhibits Ras2p; binds to and inhibits GTP-bound Ras2p at the endoplasmic reticulum (ER)
YPL095C	EEB1	-0.6652827	-0.0443837	14.98935817	Acyl-coenzymeA:ethanol O-acyltransferase; responsible for the major part of medium-chain fatty acid ethyl ester biosynthesis during fermentation

Lists of mRNAs enriched (Supplementary Table S3) or depleted (Supplementary Table S4) from the polysomal fractions of ΔES27Lb cells along with their description, taken from the SGD (*S. cerevisiae* genome database; ref. 4).

Supplementary References

1. Huang da, W., Sherman, B.T. and Lempicki, R.A. (2009) Systematic and integrative analysis of large gene lists using DAVID bioinformatics resources. *Nature protocols*, **4**, 44-57.
2. Huang da, W., Sherman, B.T. and Lempicki, R.A. (2009) Bioinformatics enrichment tools: paths toward the comprehensive functional analysis of large gene lists. *Nucleic Acids Res*, **37**, 1-13.
3. Cox, J., Hein, M.Y., Luber, C.A., Paron, I., Nagaraj, N. and Mann, M. (2014) Accurate proteome-wide label-free quantification by delayed normalization and maximal peptide ratio extraction, termed MaxLFQ. *Molecular & cellular proteomics : MCP*, **13**, 2513-2526.
4. Engel, S.R., Dietrich, F.S., Fisk, D.G., Binkley, G., Balakrishnan, R., Costanzo, M.C., Dwight, S.S., Hitz, B.C., Karra, K., Nash, R.S. *et al.* (2014) The reference genome sequence of Saccharomyces cerevisiae: then and now. *G3 (Bethesda)*, **4**, 389-398.
